# miR-F4-C12 Functions on the Regulation of Adipose Accumulation by Targeting PIK3R1 in Castrated Male Pigs

**DOI:** 10.3390/ani11113053

**Published:** 2021-10-26

**Authors:** Qiao Xu, Jie Chen, Ximing Liu, Yabiao Luo, Tianzuo Wang, Meiying Fang

**Affiliations:** 1 National Engineering Laboratory for Animal Breeding, MOA Laboratory of Animal Genetics and Breeding, Department of Animal Genetics and Breeding, College of Animal Science and Technology, China Agricultural University, Beijing 100193, China; xuqiao987@163.com (Q.X.); chenjie0710@nibs.ac.cn (J.C.); lximing2018@163.com (X.L.); yabiaoluo2021@163.com (Y.L.); wangtianzuozz@163.com (T.W.); 2 Jiang Xi Province Key Lab of Genetic Improvement of Indigenous Chicken Breeds, Institution of Biological Technology, Nanchang Normal University, Nanchang 330029, China

**Keywords:** pig, microRNA, adipose accumulation, miR-F4-C12, PIK3R1

## Abstract

**Simple Summary:**

MicroRNAs play crucial roles in regulating adipogenesis and fat storage; their role in regulating castrated male pig adipose growth is worth elucidating. Four nine-fold differentially expressed miRNAs were selected to investigate their functions on the regulation of adipose development based on our previous study. In 3T3-L1 cells and backfat tissues of castrated and intact male pigs, miR-F4-C12 was identified as involved in the adipose development using qRT-PCR and oil O staining. PIK3R1 was proposed by the TargetScan, miRDB and starBase as a target of miR-F4-C12 and verified through a dual-luciferase reporter assay and Western blot. These results revealed that miR-F4-C12 may regulate adipose accumulation in castrated male pigs by targeting PIK3R1. Our data provide a valuable foundation to understand the molecular mechanisms involved in adipose tissue metabolism to castration-induced sex hormone deficiency.

**Abstract:**

MicroRNAs (miRNAs) constitute small regulatory molecules for a wide array of biological activities (18~24 nucleotides in length), including adipogenesis and adipose deposition. Their effect is, however, incompletely defined in inducing fat accumulation in castrated male pigs. Based on our study, four nine-times miRNAs were selected to examine their functions in adipose formation activities. In 3T3-L1 cells and backfat tissues of castrated and intact male pigs, miR-F4-C12 was identified as a factor in adipose development utilizing quantitative real-time PCR (qRT-PCR). Further, miR-F4-C12 was identified to promote fat development, suggesting that miR-F4-C12 was involved in adipogenesis. Moreover, phosphoinositide-3-kinase regulatory subunit 1 (PIK3R1) was proposed by the TargetScan, miRDB and starBase as a target of miR-F4-C12 and verified through a two-luciferase reporter assay. The over-expression of miR-F4-C12 dramatically decreases the PIK3R1 protein level in 3T3-L1 cells. The mRNA and protein levels of PIK3R1 in castrated pigs are reduced relative to intact pigs, providing further evidence that PIK3R1 is involved in regulating adipose accumulation. These results suggest that miR-F4-C12 involves adipose development and may regulate subcutaneous adipose tissue accumulation by targeting PIK3R1 in castrated male pigs.

## 1. Introduction

Castration has been widely reported to induce obesity in male pigs because it totally eliminates the secretion of testosterone, which improves the flavor of male pig meat; it also results in body fat deposition [1]. When castrated male Göttingen minipigs were compared to intact male Göttingen minipigs, the fat percentage increased by approximately 24%, while the lean percentage decreased from 90.7 to 67.0 [1]. Recent studies have also demonstrated that in castrated male pigs, testosterone deprivation can considerably enhance fat formation (carcass fat percentage increased by 7%) [2]. As is well recognized, testosterone plays a critical role in regulating human and animal adipose deposition regulation, but the molecular mechanisms behind this relationship remain unknown.

miRNAs are a family of non-coding short RNAs (18–24 nucleotides in length) that regulate gene expression by lowering the quantities of target messenger RNA or blocking translation [3]. Previous research has revealed that miRNAs such as miR-143 [4,5], miR-21 [6,7] and miR-27 [8,9] were involved in the regulation of adipogenesis and obesity in mammals and humans [10,11,12]. Numerous miRNAs have been discovered in pigs, involved in the control of preadipocyte differentiation [13,14,15], adipogenesis [16,17] and fat deposition [18]. Additionally, miRNA expression patterns in adipose tissues of several pig breeds [19,20] or castrated and intact male pigs [21,22] were acquired through high-throughput sequencing. For example, in the backfat of Large White and Chinese Meishan pigs, 66 known and 140 new miRNAs were found. A total of 18 differently expressed miRNAs were identified as candidates in the subcutaneous adipose tissue of castrated and undamaged male pigs [22]. Our earlier investigation demonstrated that 177 miRNAs were differentially expressed in the backfat tissues of castrated and intact full-sib male pigs, including miR-F4-C12, whose expression level varied by more than 13.21-fold between entire and castrated male pigs [21]. In pigs, miR-F4-C12 is a novel miRNA that is a homolog of the mammalian miR-29b. MiR-29b increased adipogenesis in human adipose tissue-derived stromal cells (ADSCs) through boosting SP1-mediated suppression of tumor necrosis factor-alpha (TNF-α) [23]. The above indicates that miR-F4-C12 is a more likely candidate to regulate the fat gain process induced by castration.

Despite mounting evidence showing that miRNAs have a role in regulating adipogenesis and fat storage, their role in regulating adipose growth in castrated male pigs remains unknown. The function of miR-F4-C12 in controlling fat accumulation in 3T3-L1 cells and backfat tissues was studied in this study. Our findings contribute significantly to our understanding of adipose tissue metabolism’s molecular mechanisms in response to castration-induced sex hormone deprivation.

## 2. Materials and Methods

### 2.1. Animals and Sample Collection

This study used five pairs of full male sibs from Pietrain sire x Landrace dam crossed pigs. Male pigs were castrated (F3) and intact (F4) as previously described [21]. The paired male pigs had similar birth weights. One pig in each pair was castrated at the age of 1 week. The backfat tissues were collected from the 6th–7th rib of castrated and intact male pigs at 23 weeks from birth. The backfat thickness is significantly different between the castrated and intact male pigs (*p*-value < 0.01). More details about phenotype information are described in Table S1. Backfat tissue samples were taken and immediately frozen in liquid nitrogen, before being stored at −80 °C. 

### 2.2. Cell Differentiation and Transfection

At 5% CO_2_ and 37 °C, 3T3-L1 cells were maintained in Dulbecco’s modified Eagle’s medium (DMEM, Gibco, Grand Island, NY, USA) supplemented with 10% fetal bovine serum (FBS, Gibco, Grand Island, NY, USA) and 1% penicillin/streptomycin (Gibco). At 2 days post-confluence (defined as day 0), cells were differentiated by adding DMEM containing 10% FBS, 0.5 mM 3-isobutyl-1-methylxanthine, 1 µM dexamethasone and 5 µg insulin/mL. After two days, cells were cultured for another two days in media containing only 5 µg of insulin/mL. Following that, the medium was replaced every two days until the cells differentiated (10 days).

Using the Lipofectamine 2000 reagent, 3T3-L1 cells were transfected with miR-F4-C12 mimics and a negative control (NC) (Invitrogen, Carlsbad, CA, USA). After being transfected for two days, the cells were harvested for protein extraction. 

### 2.3. Oil Red O Staining

Cells were rinsed with phosphate buffer solution (PBS) and fixed for 10 min in 3.7% formaldehyde in PBS. The formaldehyde was then removed, and the area was rinsed three times with PBS before drying for 20 min at room temperature. The cells were fixed in 0.5% Oil Red for 20 min. Following staining, cells were rinsed with 60% isopropanol and imaged. 

### 2.4. RNA Preparation and qRT-PCR

Backfat tissue and 3T3-L1 cells were used to extract the miRNAs, which were extracted using the miRcute miRNA extraction kit (Tiangen, Beijing, China) according to the manufacturer’s instructions. The total RNA from the backfat tissue and 3T3-L1 cells was extracted using TRIzol reagent under the manufacturer’s recommendations (Thermo Fisher, Waltham, MA, USA). For the ensuing study, RNA preparations with an A260/A280 ratio larger than 2.0 as well as an A260/A230 ratio greater than 2.0 were used. To detect miRNA expression, the first-strand cDNA was generated from RNA samples using specific miRNA stem-loop primers and the Taqman MicroRNA Reverse Transcription Kit (ABI, Carlsbad, CA, USA). The expression of mature miRNAs was determined using RealMasterMix (SYBR Green, Tiangen, Beijing, China) per the manufacturer’s instructions. U6 was utilized to normalize the expression of miRNAs, which were purchased by RiboBio (Guangzhou, China). The amplification was carried out using the BIORAD Real-Time PCR equipment (BiaRad, Hercules, CA, USA) and started at 95 °C for 4 min, followed by 30 s at 60 °C, followed by 20 s at 72 °C, followed by 40 cycles at 95 °C. The FastQuant RT Kit (Tiangen, Beijing, China) was used to generate cDNA from 1 µg of total RNA, which was used to detect gene expression. The protocol that came with the kit was followed precisely. The PCR amplification procedure was carried out as previously described [21]. U6, a small nuclear RNA, was an internal reference for normalization. Relative miRNA expression was calculated using the 2^−ΔΔCt^ method. First, the Cycle Threshold (CT) values of the target miRNA were normalized with the CT values of the internal reference gene for all samples and calibration samples. The calibration sample is a mixture of all individual sample cDNAs. Second, the ΔCT values of the calibration samples were used to normalize the ΔCT values of the test samples. Finally, the expression level ratio was calculated.

ΔCT(test) = CT(target, test) − CT(ref, test)

ΔCT(calibrator) = CT(target, calibrator) − CT(ref, calibrator)

ΔΔCT = ΔCT(test) − ΔCT(calibrator)

2^−ΔΔCT^ = Relative expression

The primers and sequences utilized in this work were manufactured by Sangon (Shanghai, China), and are shown in Table 1.

### 2.5. Plasmid Construction

The target sequence of the predicted miR-F4-C12 binding site includes 3′UTR of the PIK3R1 gene, which was cloned from pig genomic DNA and inserted into the psi-check2 vector (Promega, Madison, WI, USA) using AsiSI and PmeI restriction enzymes to create the PIK3R1-3′UTR-wt plasmid. To validate the binding of miR-F4-C12 with the PIK3R1, another primer was subsequently synthesized by Sangon (Shanghai, China) to introduce mutation of some bases at the 7bp putative binding site miR-F4-C12 in PIK3R1 (PIK3R1-3′UTR-mut). Table 1 contains a list of the primer sequences.

### 2.6. Dual-Luciferase Reporter Assay

At a density of 2 × 10^4^ cells per well, Hela cells were plated in 24-well plates (Corning, Sanford, ME, USA). The cells were then transfected using the Lipofectamine 2000 reagent (Invitrogen, Carlsbad, CA, USA) with 100 ng of PIK3R1-3′UTR-wt or PIK3R1-3′UTR-mut in combination with 40 nM miR-F4-C12 mimics or negative control (Generay, Shanghai, China). Each transfection was replicated three times. Renilla and firefly luciferase activities were determined after 36 h of transfection using the Dual-Glo Luciferase Assay System (Promega, Madison, WI, USA) in a TD-20/20 luminometer (Turner Biosystems, Sunnyvale, CA, USA), and the Renilla luciferase signal was normalized to the firefly luciferase signal.

### 2.7. Protein Isolation and Western Blot Analysis

Total protein was recovered from pig backfat and 3T3-L1 cells using an ice-cold lysis buffer containing PMSF (phenylmethylsulfonyl fluoride). The samples were centrifuged at 12,000× *g* for 30 min at 4 °C. The concentration of total protein was determined using a Braford protein assay kit (Tiangen- PA102). Sodium dodecyl sulfate-polyacrylamide gel electrophoresis (SDS-PAGE) was used to separate 30 µg of total protein and transfer it to polyvinyl difluoride (PVDF) membranes (Millipore, Boston, MA, USA). Anti-PIK3R1 (ab86714, Abcam, Cambridge, United Kingdom) and anti-tubulin (ab15246, Abcam) primary antibodies were used to probe the membranes, followed by an appropriate secondary antibody (anti-rabbit-HRP (ab97051, Abcam) or anti-mouse-HRP (ab205719, Abcam)). Before usage, the primary antibodies against PIK3R1 and tubulin were diluted to 1:3000 and 1:5000, respectively. Secondary antibodies were diluted to a concentration of 1:5000.

### 2.8. Statistical Data Analysis

Data are presented as the means ± SDs. Significance difference between groups was identified through a one-way analysis of variance (ANOVA). Comparison of different groups was made by Duncan’s new multiple range test in R package agricolae (version 1.2-8, city, state abbr. if Canada or USA, country), R version 3.3.3 [21]. Differences were considered statistically significant if *p*-values were < 0.05.

## 3. Result

### 3.1. Identification of miRNA Expression in Backfat Tissue 

**Figure 1:**
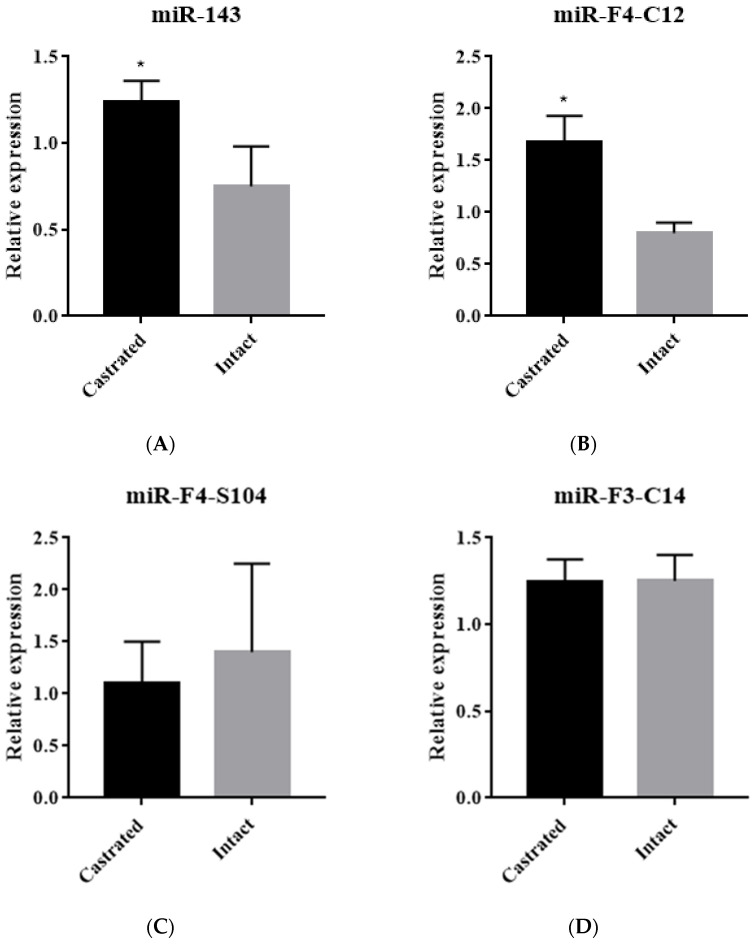

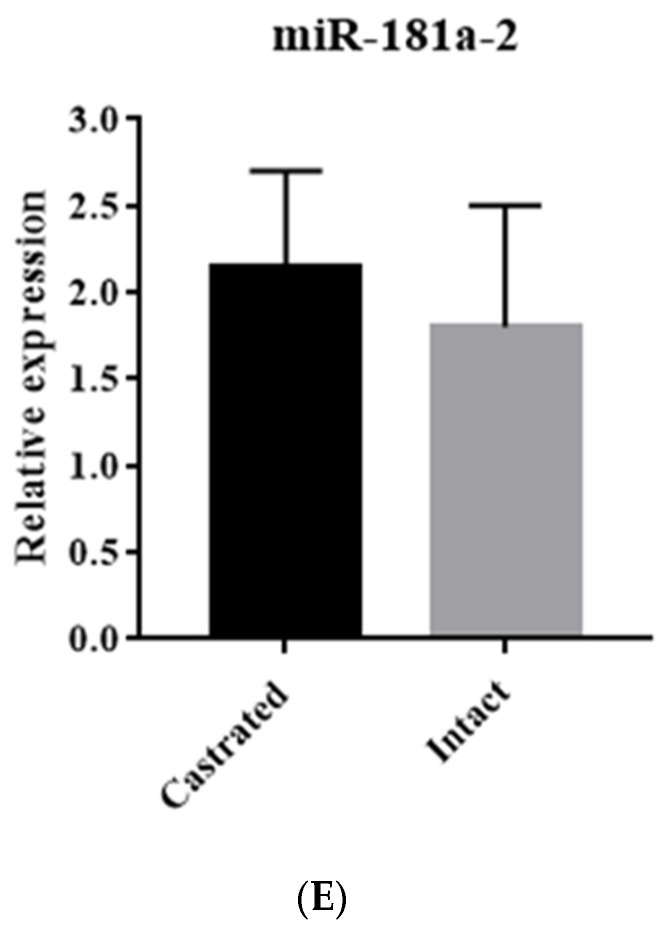
Expression levels of miRNAs in backfat tissues. (**A**) miR-143, (**B**) miR-F4-C12, (**C**) miR-F4-S104, (**D**) miR-F3-C14, (**E**) miR-181a-2. Results are shown as means ± SD of triplicate experiments. * Indicates *p* < 0.05.

Four nine-fold differentially expressed miRNAs (miR-F4-C12, miR-F3-C14, miR-F4-S104 and miR-181a-2) between castrated and intact male pigs were chosen based on the expression profile by SOLiD sequencing. Additionally, miR-143 was selected as a positive control [24]. The five miRNAs were detected in the backfat tissue of castrated and intact male pigs from five full-sib pairs using a stem-loop qRT-PCR. The results indicated that miR-143 and miR-F4-C12 expression levels were significantly elevated in castrated male pigs compared with intact male pigs (Figure 1A,B); however, miR-F4-S104, miR-F3-C14 and miR-181a-2 expression levels were not statistically different (Figure 1C–E).

### 3.2. Expression Profiles of miRNAs during Adipogenic Differentiation in 3T3 Cells

**Figure 2:**
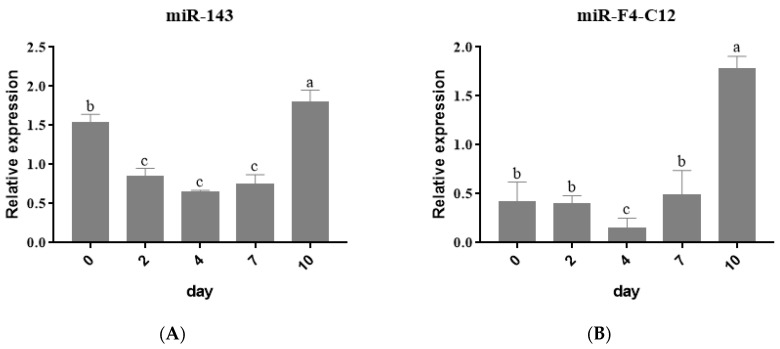

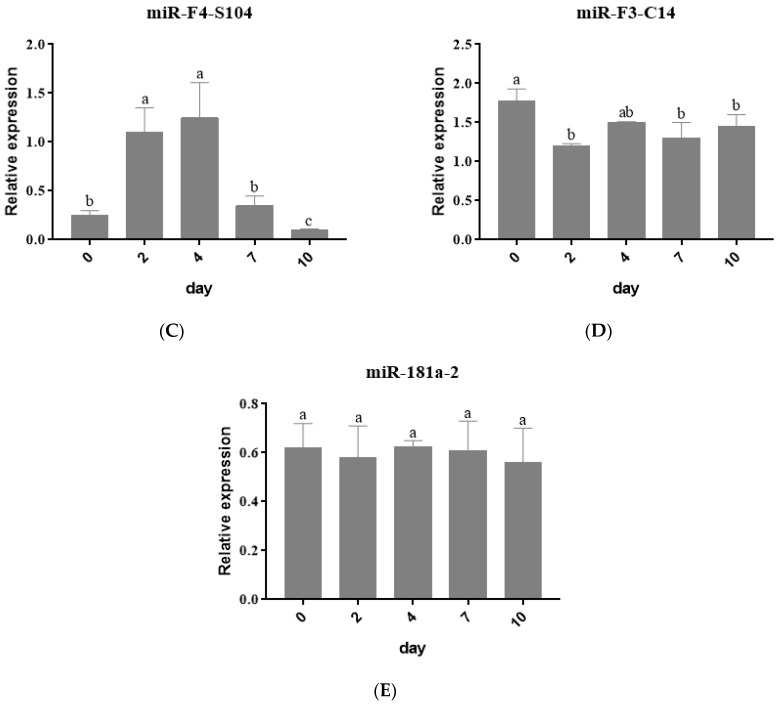
Expression levels of miRNAs in differentiation adipocytes. The expression level of five mature miRNAs at different induction times in 3T3-L1 cells. *U6* as a loading control. (**A**) miR-143, (**B**) miR-F4-C12, (**C**) miR-F4-S104, (**D**) miR-F3-C14, (**E**) miR-181a-2. Results are shown as means ± SD of triplicate measurements. Different letters indicate a significant difference (*p* < 0.05).

Following that, the expression levels of the five miRNAs were examined during the 3T3-L1 cells and were differentiated into adipocytes. Compared with the control groups, a significant buildup of lipid droplets was detected after 3T3-L1 was induced at 7 days post-confluence (Figure S1A,B), and Oil red O staining revealed the formation of lipid droplets (Figure S1C). 3T3-L1 cells were harvested at various differentiation stages, including d0, d2, d4, d7 and d10, and total RNA was extracted. The results of stem-loop qRT-PCR revealed that miR-143 expression declined from day zero (d0) to day four (d4) but increased at d7 and d10, which is consistent with earlier findings [8] (Figure 2A). The expression of miR-F4-C12 is unchanged from d0 to d2. However, after differentiation for 2 days, the miR-F4-C12 level decreased from d2 to d4 but increased dramatically from d4 to d10, and the expression at d10 is more than four-fold compared with d7 (Figure 2B). miR-F4-S104 expression increased from d0 to d4 but dropped from d4 to d10 (Figure 2C). The expression of miR-F3-C14 and miR-181a-2 is nearly identical (Figure 2D,E).

The expression pattern of miR-F4-C12 in tissues and cells was consistent with miR-143 (positive control), indicating that miR-F4-C12 may be involved in lipid accumulation.

### 3.3. miR-F4-C12 Promote the Differentiation of 3T3-L1 Cells

**Figure 3:**
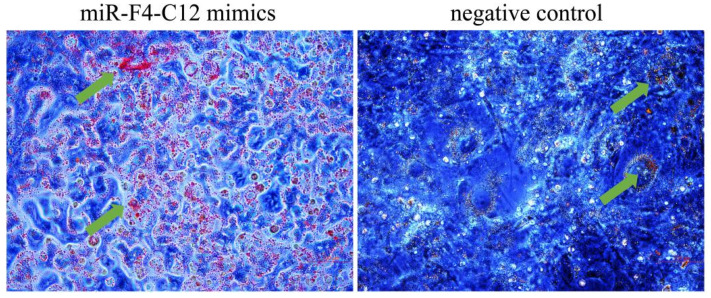
The overexpression of miR-F4-C12 promoted the differentiation of 3T3-L1 cells. Oil red staining showed increased lipid droplets in 3T3-L1 cells in the miR-F4-C12 overexpression group compared with the negative control group. Green arrows indicate red lipid droplets.

To explore whether miR-F4-C12 is involved in adipose development, 3T3-L1 cells were treated with Lipofectamine 2000 to over-express miR-F4-C12 during the culture in cells differentiation medium. After 4 days of differentiation, oil red O staining showed increased lipid droplets in 3T3-L1 cells, transfected with the miR-F4-C12 mimics compared to those treated by a negative control (Figure 3). The results showed that miR-F4-C12 promoted the differentiation of the 3T3-L1 cells, suggesting that miR-F4-C12 was involved in adipogenesis.

### 3.4. PIK3R1 Was the Direct Target of miR-F4-C12 

**Figure 4:**
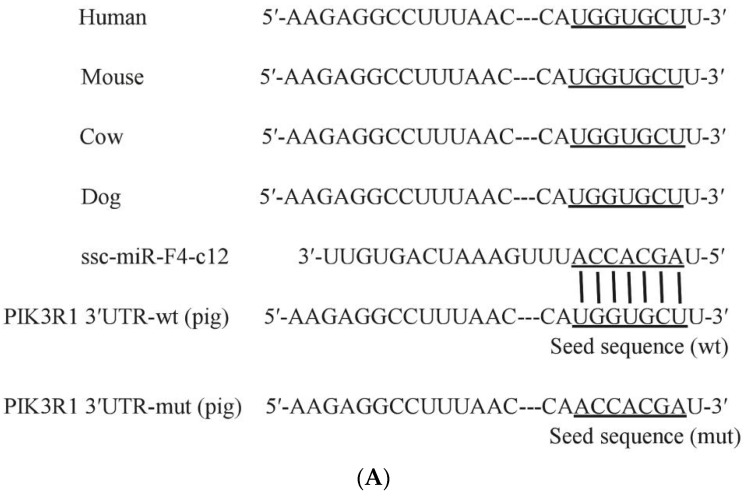

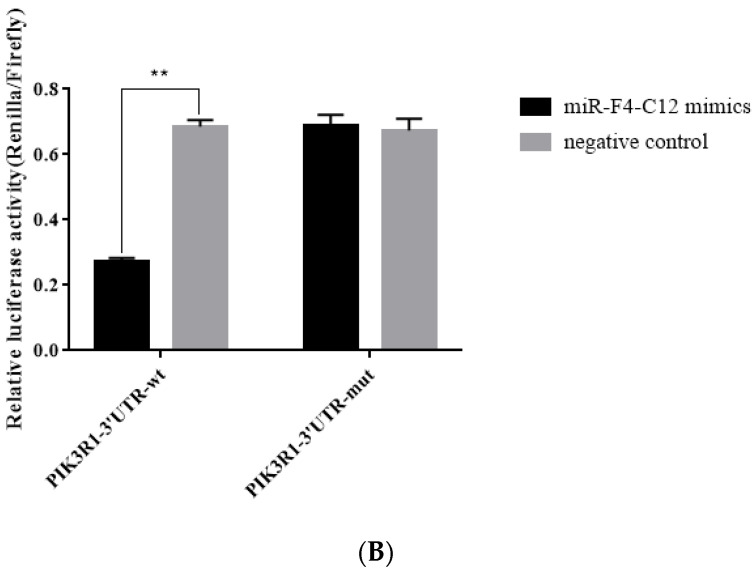
Inhibitory effect of ssc-miR-F4-C12 on PIK3R1 3′UTR using the dual-luciferase system. (**A**) PIK3R1 mRNA sequences from 5 species are aligned 5′ to 3′. Potential base pairing between ssc-miR-7134-3p (3′ to 5′) and PIK3R1 is shown in the underline region. (**B**) Cells were transfected with two plasmids containing wild type (wt) or mutant (mut) PIK3R1 3′UTR region and then treated with miR-F4-C12 mimic (black) or negative control (grey). Renilia luciferase values normalized to the Firefly luciferase values. Data represent means ± SD. ** Indicates *p* < 0.01.

To characterize genes potentially regulated by miR-F4-C12 and their roles in adipose development in animals, prediction online tools TargetScan (http://www.targetscan.org/, accessed on 3 January 2019), miRanda (http://www.microRNA.org/, accessed on 3 January 2019) and Starbase (http://starbase.sysu.edu.cn/, accessed on 3 January 2019) detected a conserved miR-F4-C12 binding site in the 3′UTR of PIK3R1 mRNA (Figure 4A). We, thus, used a dual-luciferase reporter system to test whether miR-F4-C12 could target PIK3R1 expression (Figure 4B). As shown by a dual-luciferase reporter assay in the Hela cell line (Figure 4B), co-transfection of the Hela cell line with an miR-F4-C12 mimic or negative control did not induce any difference in the luciferase activity of the reporter in cells containing PIK3R1-3′UTR-mut plasmid but induced quite different changes in luciferase activity in cells containing wild-type PIK3R1-3′UTR. When the cells with wild-type PIK3R1-3′UTR were treated with miR-F4-C12, the mimic decreased the luciferase activity compared to those treated by the negative control. The findings indicated that miR-F4-C12 may bind to the 3′UTR of PIK3R1 and that this binding is effective.

**Figure 5:**
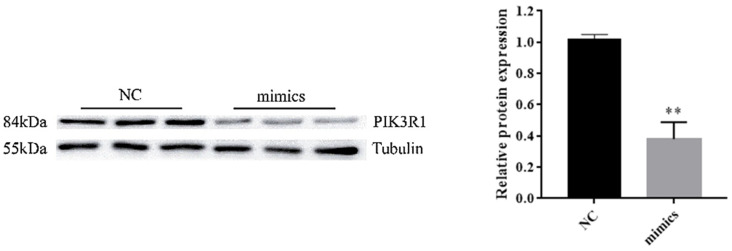
The overexpression of miR-F4-C12 repressed the protein expression of PIK3R1 in 3T3-L1 cells. Western blotting showed decreased protein expression of PIK3R1 in 3T3-L1 cells treated with miR-F4-C12 mimic compared to those treated with negative control miRNA (NC). Data are means ± SD. ** Indicates *p* < 0.01. Original western blot figures in Figures S2 and S3.

To further identify whether miR-F4-C12 regulates PIK3R1, the PIK3R1 protein level was determined in 3T3-L1 cells, which were transfected with the miR-F4-C12 mimics; then, the expression of PIK3R1 was compared with that in cells treated with negative control miRNA. As shown in Figure 5, Western blotting showed a significant decrease in PIK3R1 protein in cells treated with miR-F4-C12 mimic compared to those treated with the negative control. The results demonstrated that miR-F4-C12 could inhibit the PIK3R1 protein expression.

### 3.5. PIK3R1 Expression in Backfat Tissue

**Figure 6:**
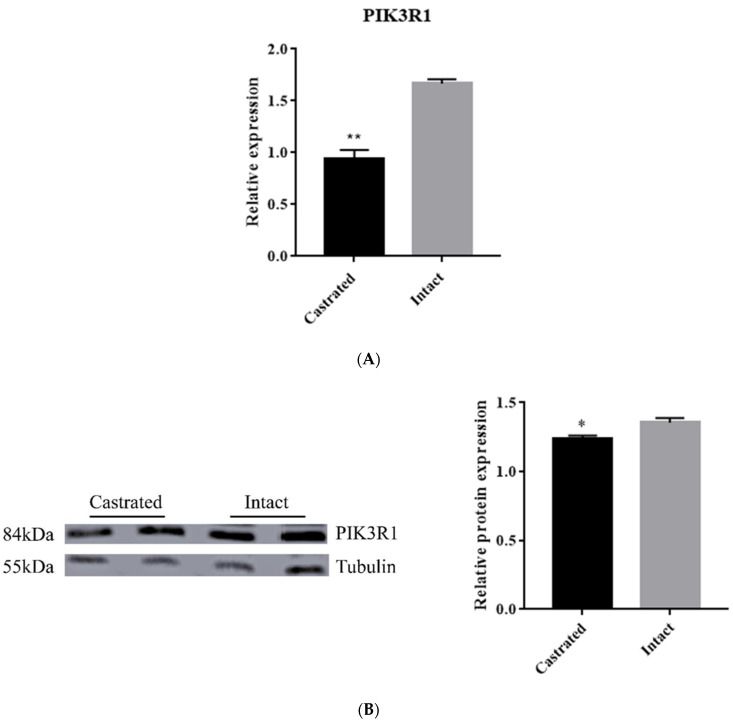
Expression of PIK3R1 protein and mRNA in backfat tissue. (**A**) mRNA expression of PIK3R1 showed significant difference in backfat tissues of castrated and intact pigs. (**B**) Western blotting showed decreased protein expression of PIK3R1 in castrated compared to intact pigs. The protein level of PIK3R1 was detected using Western blot in backfat tissue of castrated and intact pigs. Data are means ± SDs. * Indicates *p* < 0.05, ** indicates *p* < 0.01. Original western blot figures in Figure S4.

Furthermore, PIK3R1 mRNA and protein expression were determined in backfat tissue between intact and castrated pigs. The qRT-PCR data result showed that the PIK3R1 mRNA level was significantly lower (*p* < 0.01) in the castrated group than in the intact group (Figure 6A). Meanwhile, the PIK3R1 protein level was significantly decreased in the castrated group compared with that in the intact group (Figure 6B). These results are consistent with the data that miR-F4-C12 down-regulates PIK3R1 protein expression in 3T3-L1 cells. Taken together, these results revealed that miR-F4-C12 may promote adipose accumulation in castrated male pigs by inhibiting PIK3R1.

## 4. Discussion

In commercial pork production, castration in the male pig is a standard procedure to reduce boar taint, but it increases the deposit of adipose tissue. However, the molecular mechanism of the increased adipose tissues in castrated pigs is still unclear. miRNAs have recently been reported as a crucial regulatory factor involved in adipogenesis and lipid metabolism in mammals [25,26]. Previous research by our group employed a high-throughput SOLiD sequencing technology to identify and characterize miRNA expression in backfat from intact and castrated full-sib male pigs [21]. The biochemical process driving the increase in adipose tissue in castrated pigs, on the other hand, remains unknown. In this study, four microRNAs (miR-F4-C12, miR-F3-C14, miR-F4-S104 and miR-181a-2) were chosen because they showed a greater than nine-fold difference in expression between castrated and intact male pigs. These miRNAs were investigated for their potential contribution to the accumulation of adipose tissue caused by castration. The miR-F4-C12 expression was significantly higher in the castrated group than in the intact group (Figure 1B) and was increased considerably when 3T3-L1 cells were differentiated from d4 to d10 (Figure 2B), which was consistent with the expression pattern of miR-143 (Figure 1A and Figure 2A). However, this conclusion does not agree with the data obtained using SOLiD sequencing (our previous study). There are likely some flaws in the SOLiD sequencing process, which would explain this. The miR-F4-C12 is a novel miRNA in pigs and is a homolog of mammalian miR-29b. Recent studies have shown that the expression of miR-29a/b/c decreases between days one and three and then increases between days three and nine during 3T3-L1 differentiation [27], which is consistent with our findings. 

Furthermore, miR-29b has been shown to promote adipogenesis via enhancing the SP1-mediated suppression of tumor necrosis factor (TNF) during the normal adipogenic differentiation of ADSCs [23] and to be implicated in regulating lipid metabolism by reducing SCAP/SREBP-1 expression [28]. Interestingly, miR-F4-C12 was further identified to promote 3T3-L1 cells differentiation (Figure 3). Our findings showed that miR-F4-C12 is a critical regulator of fat formation and may regulate fat accumulation in castrated pigs, supported by previous research.

To investigate the role of miR-F4-C12 in the regulation of fat deposition in greater depth. PIK3R1 is a direct target of miR-F4-C12, according to the results of a dual-luciferase reporter assay and Western blot analysis (Figure 4 and Figure 5). PIK3R1 mRNA and protein levels were found to be lower in the castrated male pigs’ backfat tissue (Figure 6), but miR-F4-C12 mRNA levels were found to be greater in the castrated male pigs’ backfat tissue (Figure 1B). More importantly, when the miR-F4-C12 mimics were transfected into the cells, the protein expression of the PIK3R1 was shown to be lower than when the negative control miRNA was transfected into the cells (Figure 5). Collectively, these findings imply that miR-F4-C12 could inhibit PIK3R1 protein expression. PIK3R1 (also called p85a) is a critical component of the phosphatidylinositol 3-kinase (PI3K) signaling pathway. *PI3K* is required for the proper function of various tissue-specific biological processes, including adipogenesis [29]. After three months on a high-fat diet, the PIK3R1 knockout (PIK3R1^−/−^) mice gained significantly more body weight and white adipose tissue mass than the wild-type mice, and adipocyte size was significantly greater in the high-fat PIK3R1^−/−^ group than in the high-fat wild-type group [30]. The above findings corroborated our findings that increasing miR-F4-C12 expression lowered PIK3R1 protein expression and ultimately increased fat storage in castrated male pigs. Additionally, the researchers discovered a unique function for PIK3R1 in that glucocorticoids increased PKA levels in the lipid droplet, hence promoting lipolysis [31]. The elimination of PIK3R1 in white adipose tissue impairs the glucocorticoids’ ability to stimulate lipolysis, resulting in hypertriglyceridemia and fatty liver [31]. As a result, we hypothesize that PIK3R1 may play a critical role in the adipose accumulation of castrated male pigs.

## 5. Conclusions

Collectively, our results show that the increase in ssc-miR-F4-C12 levels, caused by castration, results in a decrease in PIK3R1 protein levels. Our data indicated that miR-F4-C12 maybe influence adipose accumulation in castrated male pigs through modulating PIK3R1. Therefore, we conjecture that PIK3R1 may play a critical role in obesity and lipidosis associated with low testosterone. However, further studies will be necessary to decipher the molecular mechanisms underlying the PIK3R1 function.

## Figures and Tables

**Table 1:** The sequence of the primers used in this study. RT: reverse transcription primer. The underlined sequence was the mutation site.

Primer Name	Primer Sequence (5′-3′)	Application
miR-F4-C12	RT: CTCAACTGGTGTCGTGGAGTCGGCAATTCAGTTGAGAACACTGA	qRT-PCR
	F: TCTCGCACGC TAGCACCATT	
	R: CTCAACTGGTGTCGTGGAGTC	
miR-F3-C14	RT: CTCAACTGGTGTCGTGGAGTCGGCAATTCAGTTGAGCGCCAATA	qRT-PCR
	F: GGG TAGCAGCACGTAAATATT	
	R: CTCAACTGGTGTCGTGGAGTC	
miR-F4-S104	RT: CTCAACTGGTGTCGTGGAGTCGGCAATTCAGTTGAGTTCAGTCA	qRT-PCR
	F: GGG CATCTGTGGGATTATGA	
	R: CTCAACTGGTGTCGTGGAGTC	
miR-181a-2	RT: CTCAACTGGTGTCGTGGAGTCGGCAATTCAGTTGAGAACTCACC	qRT-PCR
	F: TCTCGCACGC AACATTCAACGCTG	
	R: CTCAACTGGTGTCGTGGAGTC	
miR-143	RT: CTCAACTGGTGTCGTGGAGTCGGCAATTCAGTTGAGGAGCTACA	qRT-PCR
	F: TCTCGCACGCTGAGATGAAGC	
	R: CTCAACTGGTGTCGTGGAGTC	
PIK3R1	F: GACTTGCCCCACCACGAT	qRT-PCR
	R: AGTGCTTGACCTCGCCGT	
PIK3R1-wt-3′ UTR	F: ATTGCGATCGCGCCACCTGCTCAAGTTCA	Luciferase reporter
	R: ATTGTTTAAACGTTCCCCAAAGTGTTCCTCT	
PIK3R1-mut- 3′ UTR	F: ATTGCGATCGCGCCACCTGCTCAAGTTCA	Luciferase reporter
	R: ATTGTTTAAACGCTTCTGAAAGCATGAACATCGTGGTTGGTGAAAGGCCTCTTTG	

## Supplementary Materials

The following are available online at https://www.mdpi.com/article/10.3390/ani11113053/s1, Table S1: Information of experimental male pigs, Figure S1: 3T3-L1 cells differentiation. Figures S2–S4: Original western blot figures.
