# Supplementary material for: miR-F4-C12 Functions on the Regulation of Adipose Accumulation by Targeting PIK3R1 in Castrated Male Pigs
Source: Animals (Basel). 2021 Oct 26;11(11):3053. doi: 10.3390/ani11113053 (PMC8614499; doi:10.3390/ani11113053)
Supplement: Supplementary file 1 [file animals-11-03053-s001.zip › animals-1355629-supplementary.pdf]

animals

MDPI

Table S1. Information of experimental male pigs [21].

Items	Intact pigs (n = 3)	Catrated pigs (n = 3)	*p*-value
Birth weight(kg)	1.61 ± 0.02	1.62 ± 0.01	0.97
Weaning weight(kg)	8.35 ± 0.45	8.15 ± 0.15	0.71
Carcass weight(kg)	106 ± 5.00	104 ± 1.00	0.73
Backfat thickness(mm)	22.6 ± 0.03	27.72 ± 0.23	0.002

Note: Three pairs of full sibs were slaughtered at 23 weeks. Carcass weight and backfat thickness were measured at 23 weeks. Date are presented as mean ± SE.

A

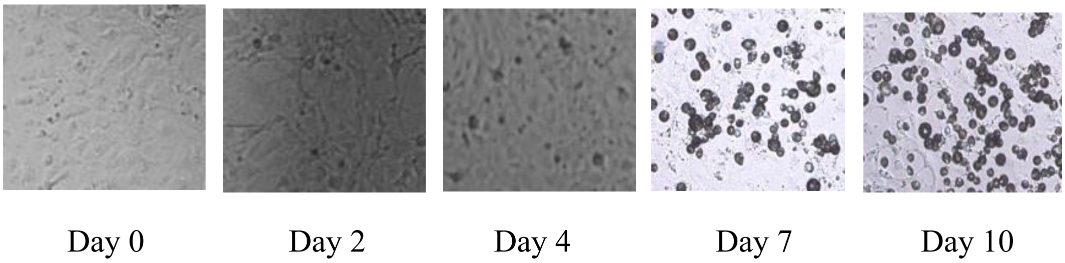

B

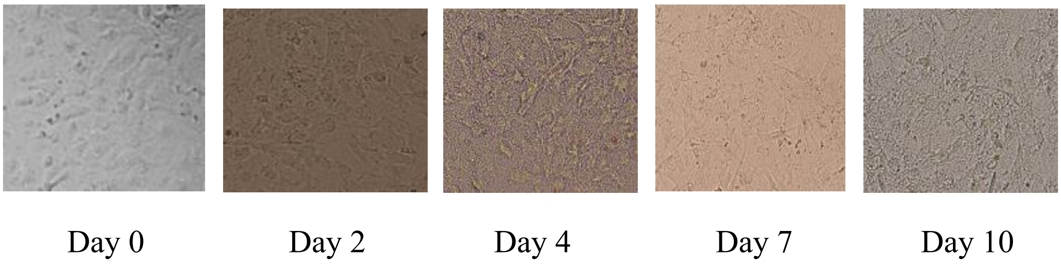

C

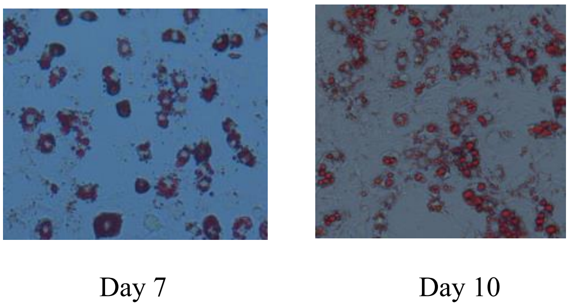

Figure S1. 3T3-L1 cells differentiation. (A) 3T3-L1 preadipocytes induced to differentiation after treated with 3-isobutyl-1-methylxanthine, dexamethasone and insulin. (B) 3T3-L1 preadipocytes cultured under normal condition. (C) Cells were fixed and stained with Oil Red O on day 7 and 10 after induced to differentiation.

The original western blot figures:

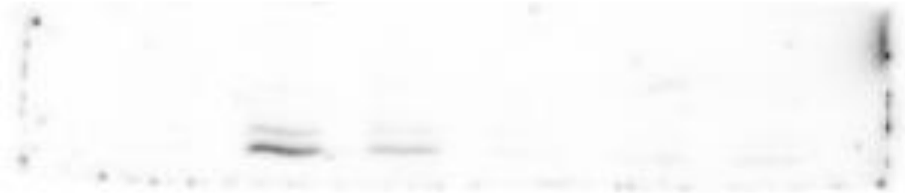

Figure S2. The original western blot of PIK3R1 protein in 3T3-L1 cells transfected with miR-F4-C12 mimics and NC (No Duplicates).

A

B

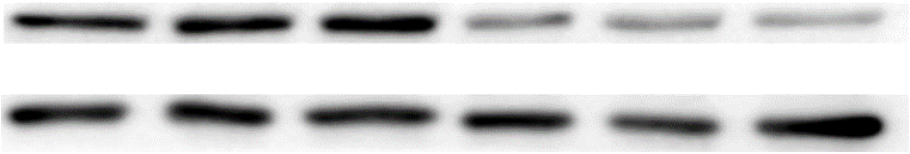

Figure S3. The original western blot of PIK3R1 (A) and Tubulin (B) protein in 3T3-L1 cells transfected with miR-F4-C12 mimics and NC (Three Duplicates, additional experiment).

A

B

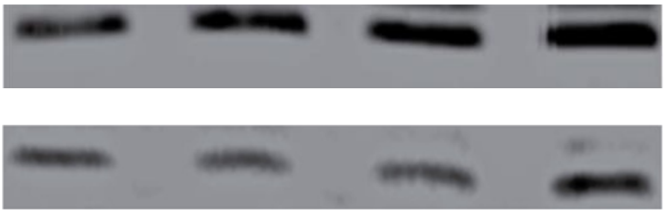

Figure S4. The original western blot of PIK3R1 (A) and Tubulin (B) protein in backfat tissue of castrated and intact pigs (Only Two Duplicates).
